# Incidental Grade 2 Appendiceal Neuroendocrine Tumor Presenting as Acute Appendicitis in a Young Adult Male Patient

**DOI:** 10.7759/cureus.83609

**Published:** 2025-05-06

**Authors:** John K Appiah, Richeal Asante, Evans Donneyong, Sreehari Cherukuri

**Affiliations:** 1 Internal Medicine, Geisinger Health System, Wilkes-Barre, USA; 2 Internal Medicine, Tamale Teaching Hospital, Tamale, GHA; 3 Integrative Medicine, Geisinger Health System, Wilkes-Barre, USA

**Keywords:** appendiceal neuroendocrine tumor, ct evaluation acute appendicitis, incidental diagnosis, uncomplicated appendicitis, young adult male

## Abstract

Neuroendocrine tumors (NETs) are epithelial neoplasms with neuroendocrine differentiation that can arise throughout the gastrointestinal tract, including the appendix. Appendiceal NETs are rare neoplasms that frequently present as or mimic acute appendicitis. We report the case of a 28-year-old male patient who presented with right lower quadrant abdominal pain and was found to have early acute appendicitis on imaging. He underwent laparoscopic appendectomy, and final histopathology revealed a 0.5 cm well-differentiated Grade 2 NET at the tip of the appendix. The tumor was confined to the submucosa, with negative margins and no lymphovascular invasion. Immunohistochemistry was positive for chromogranin and synaptophysin, and the Ki-67 proliferation index was approximately 5%. Given the tumor’s small size, complete resection, and absence of high-risk features, no further surgery was performed. The patient was referred to oncology for multidisciplinary follow-up. This case highlights the importance of histologic grading and proliferation index in the postoperative evaluation of appendiceal NETs and supports the need for routine histopathologic analysis in all appendectomy specimens.

## Introduction

Neuroendocrine tumors (NETs) are epithelial neoplasms arising from neuroendocrine cells located throughout the body, particularly in the gastrointestinal tract and lungs [1]. NETs are relatively rare, but their reported incidence has been steadily increasing over the past several decades, possibly due to improvements in diagnostic techniques and greater clinical awareness [2]. Appendiceal NETs are the most common malignancy of the appendix, accounting for approximately 0.2% to 0.5% of appendectomy specimens [3,4].

These tumors are frequently diagnosed incidentally following appendectomy performed for presumed acute appendicitis [3]. Most appendiceal NETs arise at the tip of the appendix and are small, well-differentiated lesions with excellent long-term prognosis [4]. However, tumors larger than 2 cm, those involving the base of the appendix, or those demonstrating lymphovascular invasion or mesoappendiceal extension may warrant more extensive surgical resection, such as a right hemicolectomy [5].

Tumor grade, based on mitotic activity and Ki-67 proliferation index, is increasingly recognized as a critical prognostic factor [6]. According to the World Health Organization (WHO) classification system, NETs are categorized into Grades 1 to 3 based on proliferative activity [7]. Recent guidelines from the European Neuroendocrine Tumor Society (ENETS) emphasize individualized management strategies based on tumor size, location, grade, and invasion characteristics [8]. Early identification and accurate grading of appendiceal NETs are essential for appropriate management and long-term surveillance planning [9]. Here, we present a case of an incidental Grade 2 appendiceal NET discovered during surgery for presumed acute appendicitis in a young adult male patient.

## Case presentation

**Figure 1:**
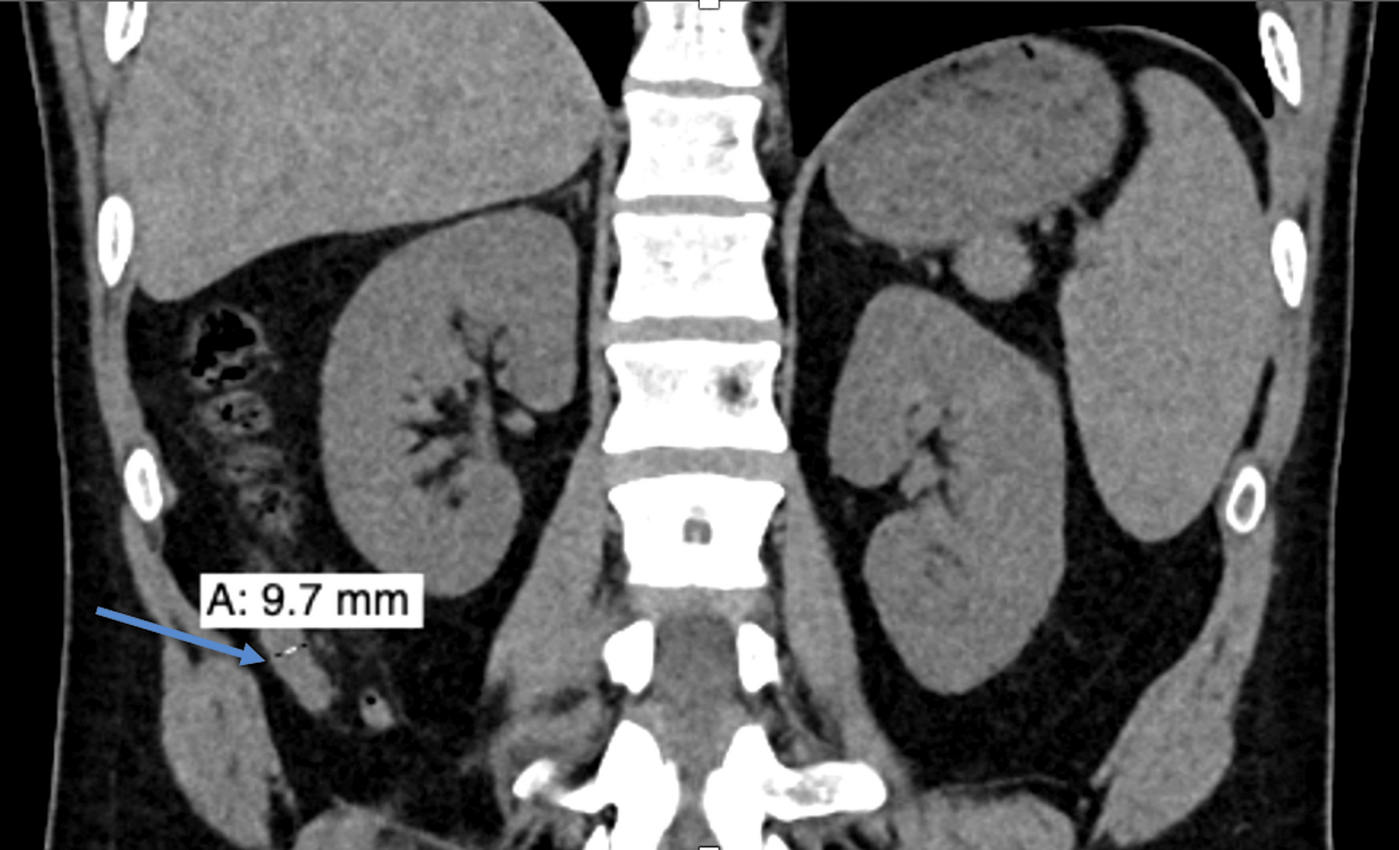
Coronal non-contrast computed tomography (CT) scan of the abdomen showing an enlarged appendix (arrow) Coronal view of a non-contrast CT scan of the abdomen demonstrating an enlarged appendix measuring 9.7 mm in maximal diameter, located in the right lower quadrant. Periappendiceal fat stranding is present, consistent with early acute appendicitis. The arrow indicates the location of the enlarged appendix.

A 28-year-old male patient with no significant past medical history presented with a three-day history of right lower quadrant abdominal pain. He denied fever, chills, nausea, vomiting, or gastrointestinal bleeding. Physical examination revealed localized tenderness at McBurney's point. Laboratory evaluation showed leukocytosis with a white blood cell count of 12.65 x 10⁹/L (normal range: 4.0 to 10.8 × 10⁹/L). A non-contrast computed tomography (CT) scan of the abdomen (Figure 1) showed thickening of the distal appendix with surrounding fat stranding, consistent with early acute appendicitis.

The patient underwent an uncomplicated laparoscopic appendectomy. Intraoperatively, the appendix appeared inflamed but without evidence of perforation or mass. Histopathologic evaluation revealed a 0.5 cm well-differentiated NET located at the tip of the appendix. The tumor was confined to the submucosa with negative margins and no lymphovascular invasion. Immunohistochemical staining was positive for synaptophysin and chromogranin. The Ki-67 proliferation index was approximately 5%, consistent with a Grade 2 NET.

The patient recovered uneventfully postoperatively. Given the small tumor size, negative margins, and absence of high-risk features, further surgical intervention was not recommended. The patient was referred for multidisciplinary follow-up with oncology.

## Discussion

Appendiceal NETs are frequently diagnosed incidentally following surgery for presumed acute appendicitis [3]. These tumors may contribute to appendicitis by causing luminal obstruction or localized inflammation. Most NETs smaller than 1 cm and confined to the tip of the appendix are adequately managed with appendectomy alone [4]. However, tumor size, location, histologic grade, lymphovascular invasion, and mesoappendiceal extension must be carefully assessed to determine the need for further surgical intervention [5].

Management decisions must be individualized. For tumors greater than 2 cm, or for those between 1 and 2 cm with adverse features such as lymphovascular invasion, mesoappendiceal invasion greater than 3 mm, high mitotic rate, or positive margins, a completion right hemicolectomy is generally recommended [5]. Right hemicolectomy allows for regional lymph node evaluation and removal, which is important because metastatic spread correlates strongly with tumor size and grade.

Although our patient’s tumor was small (0.5 cm) and confined to the submucosa, the Ki-67 index of 5% classified it as a Grade 2 NET [7]. Grade 2 tumors are associated with a higher risk of recurrence and metastatic potential compared to Grade 1 tumors [6]. Consequently, even in cases where further surgery is not indicated, careful multidisciplinary follow-up is essential. Surveillance typically includes periodic imaging and monitoring of NET markers, although formal surveillance protocols for appendiceal NETs remain less standardized compared to other gastrointestinal NETs.

The overall prognosis for small, well-differentiated appendiceal NETs remains excellent, with five-year survival rates exceeding 90% [2,9]. Nonetheless, individualized risk assessment based on tumor size, grade, and other histopathologic features remains crucial for optimizing patient outcomes. Multidisciplinary team input involving surgery, oncology, pathology, and radiology specialists is fundamental for guiding the management and surveillance of these rare tumors.

## Conclusions

This case highlights that even small, incidentally discovered appendiceal NETs may require tailored follow-up based on the histologic grade and proliferative index. Routine histopathologic examination of all appendectomy specimens and multidisciplinary coordination are essential components for optimizing patient outcomes.
